# Enhanced Visible Light Driven Photocatalytic Behavior of BiFeO_3_/Reduced Graphene Oxide Composites

**DOI:** 10.3390/nano8070526

**Published:** 2018-07-13

**Authors:** Yun-hui Si, Yu Xia, Shao-ke Shang, Xin-bo Xiong, Xie-rong Zeng, Ji Zhou, Ya-yun Li

**Affiliations:** 1 Shenzhen Key Laboratory of Special Functional Materials & Shenzhen Engineering Laboratory for Advance Technology of Ceramics, College of Materials Science and Engineering, Shenzhen University, Shenzhen 518060, China; 2161120218@email.szu.edu.cn (Y.S.); 2172341411@email.szu.edu.cn (Y.X.); 2172341466@email.szu.edu.cn (S.S.); xxbszdx@szu.edu.cn (X.X.); zengxier@szu.edu.cn (X.Z.); 2 State Key Laboratory of New Ceramics and Fine Processing, School of Materials Science and Engineering, Tsinghua University, Beijing 100084, China; zhouji@tsinghua.edu.cn

**Keywords:** BiFeO_3_, reduced graphene oxide, photocatalytic, visible light

## Abstract

BiFeO_3_/Reduced Graphene Oxide (BFO/RGO) composites have been fabricated by a simple hydrothermal method. The X-ray diffraction (XRD), scanning electron microscopy (SEM), Raman, and X-ray photoelectron spectroscopy (XPS) analysis reveal that graphene oxide was reduced in hydrothermal process and BFO/RGO composites were successfully synthesized. UV-visible absorption and photoluminescence properties show that the introduction of RGO can effectively reduce the recombination of photogenerated electron and hole pairs. Compared to the pristine BFO, the photocatalytic performance of BiFeO_3_ Graphene Oxide (BGO) composites is enhanced for the degradation of Methylene blue (MB) solution under visible light irradiation, and the result shows that the optimal amount of Graphene Oxide (GO) in the composites is 60 mg (BGO60). The excellent photocatalytic performance is mainly ascribed to improved light absorption, increased reactive sites, and the low recombination rate of electron-hole pairs. This work can provide more insights into designing advanced photocatalysts for wastewater treatment and environmental protection.

## 1. Introduction

In recent decades, with the rapid development of economic globalization, the energy crisis and environmental pollution have become the two main challenges that restrict the sustainable development of our society. The excessive exploitation and utilization of coal, oil, and other fossil fuels has brought irreversible damage to the earth’s ecosystem. Industrial wasted water, gas and residue exist widely in water, soil, and the atmosphere, which are difficult to decompose through natural processes. In addition, the rapid development of industry is stimulating the demand of energy, while conventional fossil fuels are limited and non-renewable. In 1972, A. Fujishima and K. Honda reported that hydrogen and oxygen was produced by the decomposition of water on a TiO_2_ single crystal electrode [1], this discovery confirms that clean energy hydrogen can be produced by using ultraviolet light in the sunlight, which has a profound impact on the energy crisis. As an opportunity, the era of photocatalytic technology has been opened. Semiconductor photocatalytic technology can convert solar energy into electrical energy and chemical energy [2,3,4]. Many semiconductor materials, such as TiO_2_, ZnO, Fe_2_O_3_, and CdS, have a suitable band structure as a photocatalyst [5,6,7,8]. Among them, TiO_2_ has become the most widely used photocatalyst because of its stable chemical properties, strong oxidation reduction, corrosion resistance, non-toxic, and low cost. However, TiO_2_ has a wide band gap (3.2 eV), which can only be stimulated by ultraviolet light with wavelengths less than 387 nm [9]. The energy of ultraviolet light is only 4% of the total energy of the sun, which makes the utilization of the sun light very low. In addition, TiO_2_ nanoparticles are often difficult to recycle and reuse, this also seriously restricts its application and development.

Bismuth ferrite (BiFeO_3_, BFO) is a kind of multiferroic material with ferroelectric and ferromagnetic properties at room temperature at the same time [10]. Its high ferroelectric Curie temperature and ferromagenicNéel temperature determine wide application prospects in memory, sensors, and spintronic devices. Recently, studies have shown that BFO is a new generation of photocatalyst with visible light response [11]. The experimental test shows that BFO has a narrow band gap, and its band gap energy is about 2.2 eV [12]. The absorbance spectral response range of BFO includes UV and visible light, which is expected to be an excellent visible light driven semiconductor catalyst. Furthermore, it is easy to recover due to its magnetic property at room temperature [13]. A large number of experiments show that BFO has excellent degradation ability to organic pollutants under visible light irradiation [14,15]. However, the high recombination rate of photogenerated electron-hole and low quantum yield restrict the wider application of BFO in waste water treatment industry. For the purpose of enhancing the photocatalytic activity of BFO, a lot of studies have been carried out [16,17]. The enhancement of actual photocatalytic performance depends on reducing the recombination of photo-generated electrons and holes, heightening the light absorption, and improving the ability to absorb the target molecules. The most important thing is to rapidly separate the electrons and holes excited in the conduction band, and reduce the recombination rate of the electron-hole pairs. The recombination of photogenerated electron-hole pairs is usually suppressed by creating electron or hole capture centers in the lattice of the catalyst, and the doping of foreign atoms in the BFO (A or B position) has been proved to be an effective method [18,19]. In addition, the surface deposition of noble metals and the construction of heterostructures with other semiconductor materials are also considered feasible methods to enhance the photocatalytic activity of BFO [20].

Based on the novel physical and chemical properties and broad application prospects, two dimensional (2D) materials have attracted much attention in the field of catalysis [21,22]. Graphene is one of the most popular nano structure materials with a two dimensional honeycomb lattice structure consisting of a carbon six membered ring [23]. In recent years, graphene has been used as an efficient photocatalyst to improve the efficiency of an artificial photosynthesis system [24,25]. In spite of this, graphene is nonpolar and difficult to dissolve in common polar solvents such as water and ethanol, so it is difficult to compound with other materials. As one of the important derivatives of graphene, graphene oxide (GO) is a new type of carbon material with excellent performance, with high surface area and rich functional groups on the surface [26]. Due to its rich oxygen containing functional groups on the surface, graphene oxide exhibits polarity and strong hydrophilicity, which makes it easily dissolve in an aqueous solution. On account of the serious functionalization of the conjugated network, the graphene oxide thin sheet has the characteristics of insulation. Highly conducting graphene can be obtained by reduction of GO with various reducing agents [27]. Recent research reveals that the reduction of graphene oxide by the hydrothermal method has greatly improved the electron mobility and charge capacity of graphene oxide compared with chemically reduced graphene oxide (RGO) [28,29]. More importantly, its surface oxygen-containing functional groups provide rich binding sites for the photocatalytic materials. A large number of studies have reported that many photocatalytic effect semiconductor materials are combined with reduced graphene oxide [30], and can achieve enhancement in photocatalytic performance [31,32,33,34]. This strategy is of great interest to us. Based on the reports that semiconductor/graphene composites have been able to improve the photocatalytic performance of semiconductor photocatalysts, it is generally believed that the mechanism of performance enhancement include the enhanced adsorption properties of composite materials and the formation of carbon material/semiconductor interfaces to promote electron hole pair separation. Little work has been done to systematically study the effect of graphene addition on the morphology and photocatalytic properties of BFO, and the corresponding mechanism is not clear. In our study, it is found that a small amount of GO will increase the size of BFO particles and decrease photocatalytic performance. Only a suitable amount of GO could obtain enhanced photocatalytic properties in the composites. We explain this phenomenon and expect to provide ideas for the design of new photocatalytic materials in the future. 

In this work, we synthesized BFO/RGO composites through a one step hydrothermal method, the RGO nanosheets were successfully immobilized to the surface of BFO particles. We have systematically studied the influence of the addition amount of GO on the phase, morphology, and photocatalytic properties of the BFO/RGO composite photocatalysts. The possible mechanism of enhanced photocatalytic performance is proposed.

## 2. Experimental

### 2.1. Sample Preparation

Graphene oxide was synthesized by a modified Hummer’s method, which has been reported by the literature [35]. Bi(NO_3_)_3_·5H_2_O and Fe(NO_3_)_3_·9H_2_O with 1:1 molar ration (5 mmol) were dissolved in the 10 mL nitric acid (4 M).The mixture was stirred vigorously to get a clarified solution at room temperature. 30 mL of KOH solution (12 M) was dripped into the above solution as slowly as possible. Then the GO powder of different amounts was added to the mixture and continuously stirred. The reddish brown precipitation was treated by ultrasonic treatment and transferred into a 50 mL teflon lined stainless vessel and heated at 180 °C for 24 h. The obtained samples were repeatedly washed with deionized water and absolute ethanol, and dried at 80 °C in a vacuum oven for 8 h. According to the difference in the amount of GO, the final samples are named as BGO10 (10 mg), BGO30 (30 mg), BGO60 (60 mg), BGO100 (100 mg), and BGO150 (150 mg).

### 2.2. Characterization

The synthesized samples were characterized for structural analysis using X-ray powder diffraction (D8Advance, Bruker, Karlsruhe, Germany) and chemical state analysis using X-ray photoelectron spectroscopy (Escalab 250XI, ThermoFisher Scientific, Waltham, MA, USA). The grain morphology was examined with scanning electron microscopy (SU70, Hitachi, Tokyo, Japan). Raman spectra of BGO samples were taken with a Raman Microscope (inVia, Renishaw, Gloucester, Britain). The optical characteristics using UV-visible absorption/diffuse reflectance spectroscopy on a UV-2650 (Shimadzu, Kyoto, Japan). The photoluminescence spectroscopy (F-4500, Hitachi, Tokyo, Japan) of BFO and BGO samples are operated at room temperature at the excitation wavelength at 325 nm.

### 2.3. Photocatalytic Experiment

The photocatalytic activities of the as-synthesized BFO and BGO samples were studied by the decolorization of methylene blue (MB) solution under visible light irradiation (Xe lamp, 500 W; cutoff filter, λ > 420 nm). In order to avoid the influence of thermal effect, the photocatalytic reaction is kept at room temperature by the circulating water cooling. In a typical experimental experiment, the photocatalytic reaction was performed through a 100 mL 20 mg/L MB solution suspended with a 25 mg photocatalyst in a reactor under continuous magnetic stirring, which was irradiated by xenon lamp. As a comparison, the blank samplewasmeasured without any photocatalyst and other conditions remained the same. The suspension was stirring for 30 min to reach the adsorption-desorption equilibrium in a dark environment before irradiation. Subsequently, the degradation efficiency of MB was evaluated by centrifuging the retrieved sample and measuring the intensity of the absorption peak of MB (λ = 664 nm) relative to its initial intensity (C/C_0_) using a UV–V spectrophotometer (UV-2450, Shimadzu, Kyoto, Japan). The stability of the photocatalyst was studied by washing and drying the last used sample.

## 3. Results and Discussion

### 3.1. Structure and Phase Analysis

**Figure 1:**
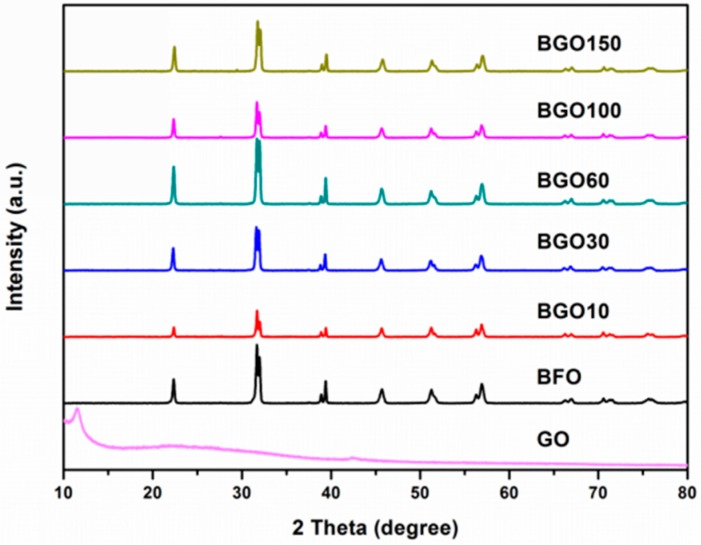
X-ray diffraction (XRD) patterns of Graphene Oxide (GO), bismuth ferrite (BFO) and BiFeO_3_ Graphene Oxide (BGO) composites.

The X-ray diffraction (XRD) patterns of the GO, BFO, and BGO composites are shown in Figure 1. The XRD patterns are in well agreement with JCPDS data (No. 86-1518), R3c space group, and the perovskite based rhombohedral structure. All of the BGO samples are indexed as pure phase BFO, without other secondary or impurities. It can be considered that a proper amount of GO introduction does not change the phase structure of BFO, and shows that the process adopted is mature and stable. There are no r-GO diffraction peaks detected in the BGO composites, this may be ascribed to the fact that the r-GO concentration is too small in the composites, leading to the disappearance of the diffraction peaks of the r-GO. The diffraction peak at 12.7 degrees can be corresponding to the (001) peak of graphene oxide, and the weak peak at 42.5 degree is indexed to the (100) peak, it is considered that the graphene oxide was fully oxidized. It is worth noting that the characteristic peak of graphene oxide at 12.7 degrees disappears in the BGO composites, and this confirms that graphene oxide was successfully reduced in the hydrothermal process. At the same time, the diffraction peak intensity of the BGO composites decreases as compared with the pure phase BFO; this should be due to the presence of reduce graphene oxide that reduces the crystallinity of the BGO composites.

**Figure 2:**
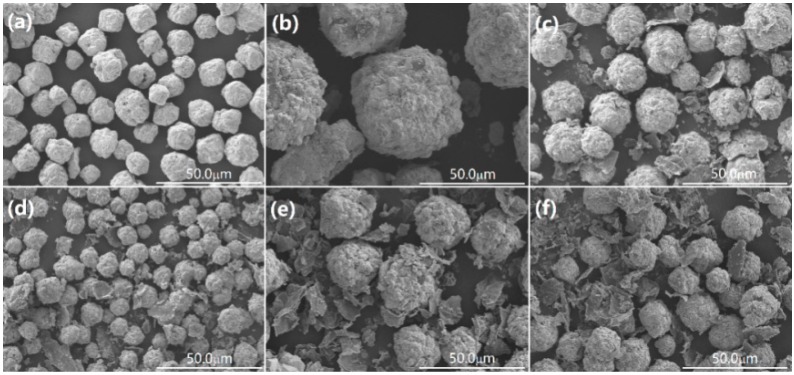
Scanning electron microscope (SEM) images of (**a**) BFO; (**b**) BGO10; (**c**) BGO30; (**d**) BGO60; (**e**) BGO 100; (**f**) BGO150.

**Figure 3:**
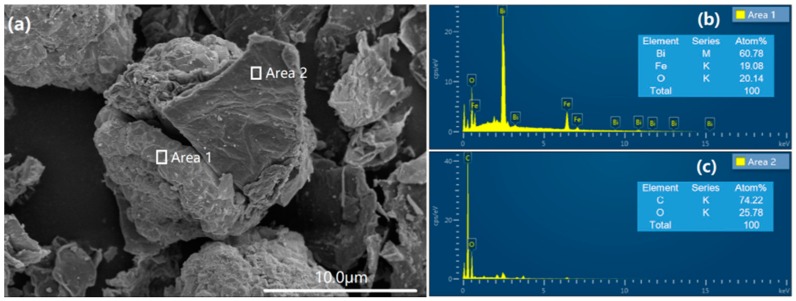
The magnification of (**a**) BGO60 composite; energy dispersive X-ray spectroscopy (EDS) spectrum of BGO60; (**b**) Area 1; (**c**) Area 2.

The morphology and microstructure of the as-synthesized BFO and BGO samples were observed by scanning electron microscopy (SEM), as shown in Figure 2a–f. Pure BFO (Figure 2a) shows a spherical morphology with a uniform particle size. Compared with pure BFO, there is a significant change in the morphology of the BFO/RGO composites due to the introduction of GO, as shown in Figure 2b–f. The layered RGO were attached to the surface of BFO particles and distributed randomly. Owing to the introduction of GO, the size of the BFO particles increased significantly, and the surface became more rough, as shown in Figure 2b. Due to the large longitudinal and transverse ratio of the graphene oxide nanoscale, the dispersion of graphene oxide in the amorphous precursor of BFO leads to the formation of the interpenetrating network of RGO in the composite of BFO and RGO [36]. The growth of BFO was limited by the network of RGO, the final particle size of the BFO was reduced compared with pure BFO. With the increase of GO content, the size of BFO the particles decreased, and BGO60 showed the smallest particle size in both BFO and BGO samples (Figure 2d). The slight introduction of GO would act as the aggregation center of BFO particles, which could be the reason for the larger size of BGO10.The photocatalyst with smaller size has a larger specific surface area and increased reactive sites, which will contribute to the enhancement of photocatalytic activity. It is noteworthy that when the GO content continues to increase, the size of the BFO particles become larger and cannot be tightly combined with the RGO layer (Figure 2e,f). Because of the high content of acidic oxygen functional groups in GO, the presence of excess GO leads to the decrease of alkalinity in an aqueous solution [37], the lower alkaline concentration leads to the rapid growth of crystal rather than nucleation, which may be the reason for the increase of particle size in BGO100 and BGO150 [12]. In order to further demonstrate the successful synthesis of BFO/RGO composites, the magnification of BGO60 was provided (Figure 3a). The typical morphology of BFO and RGO could be observed, marked as Area 1 and Area 2, respectively. The energy dispersive X-ray spectroscopy (EDS) pattern of BGO60 confirmed that the composite photocatalyst was composed of Bi, Fe, O, and C elements. As seen in the insert of Figure 3b, the atomic ratio of Bi, Fe, and O in Area 1 was close to the theoretical value of BFO. EDS results showed that only two elements of C and O were found in Area 2 (Figure 3c), indicating that reduced graphene oxide was attached to the surface of BFO. It can be summed up that the desired BFO/RGO composites are successfully synthesized during the hydrothermal process along with the introduction of appropriate amount of GO.

**Figure 4:**
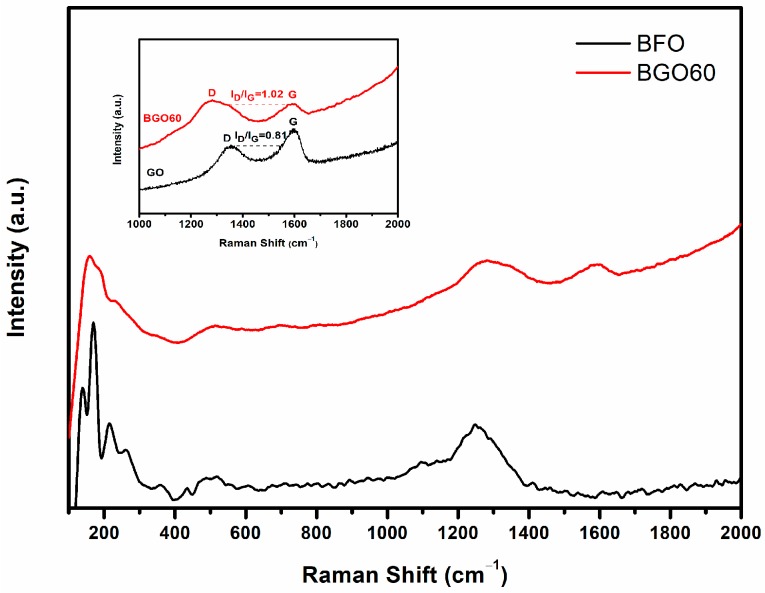
Raman spectra of BFO and BGO60. Inset shows D band and G band of BGO60 and GO.

Raman spectroscopy is an effective method for detecting the structure of materials and the vibration characteristics of molecules. The structure and structural phase transition of BFO can be determined by the position and strength of the Raman peaks. BFO has a perovskite structure with distorted trapezoid hexahedron at room temperature, called the R3c space group [38]. According to the literature, 10 atoms of the crystal cell of a perovskite structure can produce 18 optical phonon modes: 4A_1_+5A_2_+9E, A_1_ and E mode are the Raman activation modes, A_2_ mode is inactive to Raman [39]. In general, Bi-O band is corresponding to A_1_, E_1_ and E_2_ vibration modes, Fe-O band is corresponding to E_3_ and E_4_ vibration modes. In our study, 4 A_1_ modes and 8 E modes (100–700 cm^−1^) are consistent with the vibration mode in the literature [40], the Raman peaks at 137, 169, 216, and 430 cm^−1^ for BFO could be ascribed to A1-1, A1-2, A1-3, and A1-4 vibration modes, and the remaining Raman peaks at 260, 275, 305, 347, 372, 468, 526, and 610 cm^−1^ could be corresponding to E2, E3, E4, E5, E6, E7, E8, and E9 vibration modes. Raman results also confirm that the synthesized BFO is pure phase without impurity formation. As shown in Figure 4, the coexistence of BFO and RGO has been confirmed by the Raman spectra for BGO60, two discrete peaks at 1278 cm^−1^ and 1591 cm^−1^ are assigned to the D band and G band of RGO, the remaining peaks are the Raman spectrum of the perovskite structural BFO. D band represents the defects of the C atomic lattice, G band represents the stretching vibration of C atom sp2 hybrids. This can confirm that the BFO/RGO composites are successfully synthesized. In general, the intensity ratio of D peak to G peak (I_D_/I_G_) is used to characterize the defect density in graphene. The peak intensity ratio of GO is 0.81 in our study as shown in the inset of Figure 4, which is noteworthy as the corresponding value of the BGO60 is 1.02, which is higher than that of GO. It is proved that GO is reduced to RGO during the hydrothermal process, and BFO/RGO composites are successfully synthesized.

**Figure 5:**
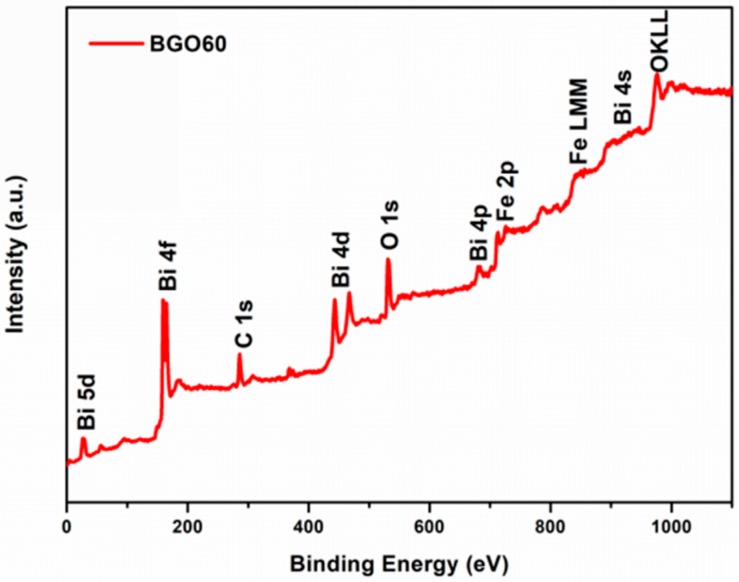
Full survey spectrum of BGO60.

**Figure 6:**
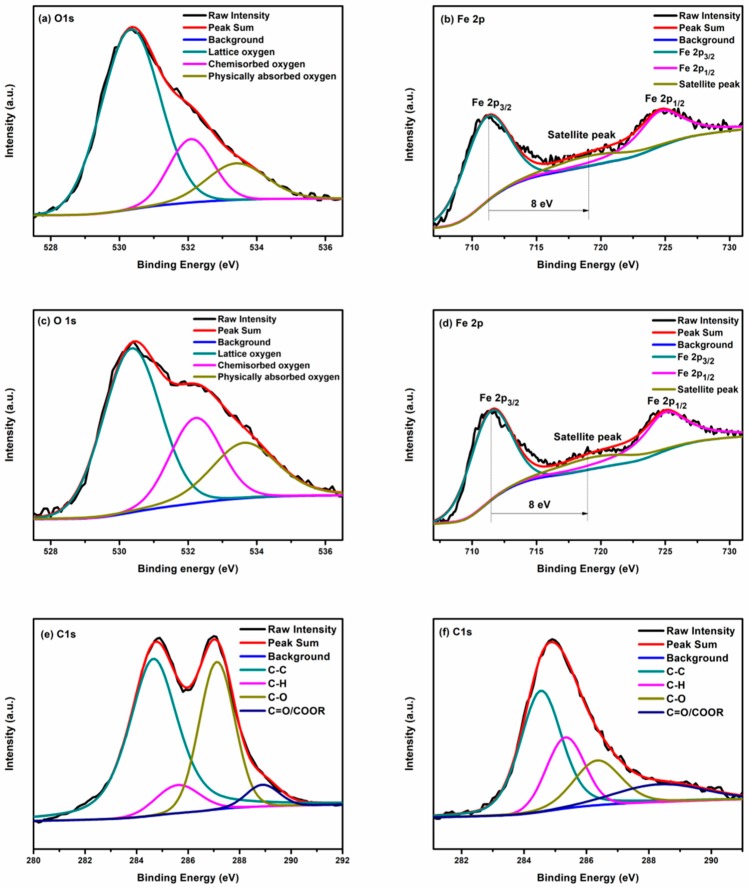
The high resolution spectrum of (**a**) O 1s for BFO; (**b**) Fe 2p for BFO; (**c**) O 1s for BGO60; (**d**) Fe 2p for BGO60; (**e**) C 1s for GO; (**f**) C 1s for BGO60.

X-ray photoelectron spectroscopy (XPS) is performed to analyze the surface chemical compositions and the oxidation state of the elements presented in the as-synthesized BFO and BGO60 samples. The full survey spectrum of the BGO60 sample is shown in Figure 5, the dominant peaks can be assigned to Bi 4f, Bi 4d, Bi 5d, Fe 2p, O 1s, and C 1s core level states, the weak peaks are attributed to Fe 2p and Bi 4s. The binding energy was obtained through correction of charging effect by C 1s peak at 284.8 eV. Figure 6a,b show the high resolution spectra of O 1s and Fe 2p core level of BFO. The binding energy of O 1s in BFO appears at 530.2, 532.1, and 533.4 eV corresponding to lattice oxygen, chemisorbed oxygen and physically absorbed oxygen, respectively. The doublet peak at 711.0 eV and 724.6 eV for BFO can be corresponding to Fe 2p_3/2_ and Fe 2p_1/2_ core level states arising from the spin-orbital splitting, respectively. The spin orbit splitting energy of Fe 2p_3/2_ and Fe 2p_1/2_ is calculated to 13.6 eV, which is consistent with the value of Fe^3+^ in the literature [41]. In addition, a satellite peak appears at 719.0 eV between Fe 2p_3/2_ orbit and Fe 2p_1/2_ orbit, about 8 eV above the Fe 2p_3/2_ orbit, and this further confirms the +3 valence state of the Fe iron in the as-synthesized BFO sample [42,43]. As shown in Figure 6c, the binding energies of O1s in BGO60 composite are observed at 530.3, 532.2, and 533.6 eV for the positions of lattice oxygen, chemisorbed oxygen and physically absorbed oxygen, respectively. Compared with BFO (Figure 6b), the peak positions of Fe 2p_3/2_, Fe 2p_1/2_ and the satellite peak in BGO60 composite are shifted towards higher binding energy, located at 711.4, 724.9 and 719.4 eV (Figure 6d), the Fe 2p slight shift might be due to the interaction with many oxygen containing groups in the RGO network. At the same time, O1s of BGO60 is also slightly shifted to high binding energy. It should attribute to the higher amount of C-O bonds in BGO60. As shown in Figure 6e, the C 1s for GO is deconvoluted into four peaks at 248.6, 285.4, 287.1, and 289.0 eV, corresponding to C-C, C-H, C-O and COOH/C=O groups, respectively. The binging energy of C 1s for BGO60 (Figure 6f) located at 284.5, 285.3, 286.3 and 288.3 eV, which could be assigned to C-C, C-H, C-O, and COOH/C=O groups, respectively. Compared with GO, the relative area percentage of C-O decreased in BGO60, while the proportion C-C increased. This change proves that GO was successfully reduced during the hydrothermal process.

### 3.2. UV-Visible Absorption and Photoluminescence Properties

**Figure 7:**
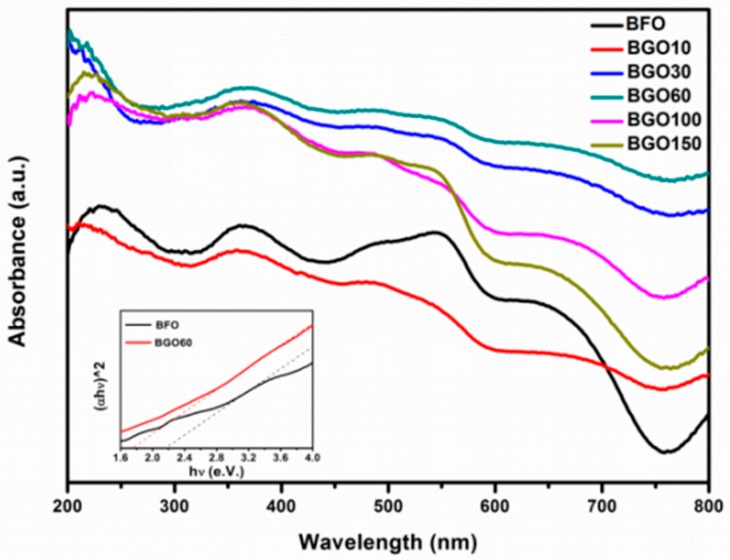
UV-visible absorption spectra of BFO and BGO samples. The inset shows the calculation of band gap.

The optical absorption of the as-synthesized BFO and BGO samples were characterized by UV-visible diffuse reflectance spectroscopy (DRS), as shown in Figure 7. BFO has a strong light absorption in the range of 200 to 550 nm and a sharp decline in absorption over 550 nm. The limited visible light absorption restricts the application of BFO as an efficient visible light photocatalyst. Compared to the pure BFO, BGO exhibited a better light absorption and can be extended to the visible light range (420–800 nm). In all of the samples, BGO60 has the most excellent optical absorption properties in both UV and visible light region. Stronger optical absorption means more photoelectron hole pairs are produced, which will effectively enhance the photocatalytic performance. The light absorption of BGO10 is relatively weak, this could be attributed to its larger particle size. As shown in the inset of Figure 7, the band gap values of BFO and BGO composites could be estimated on the basis of the equation (αhν) = A(hν − Eg)^n/2^ [11]_._ The band gap energy (Eg) of BFO and BGO60 were deduced to be 2.20 and 1.78 eV, respectively. The decrease in the band gap of the BGO composites should be due to the interaction of BFO and RGO.

**Figure 8:**
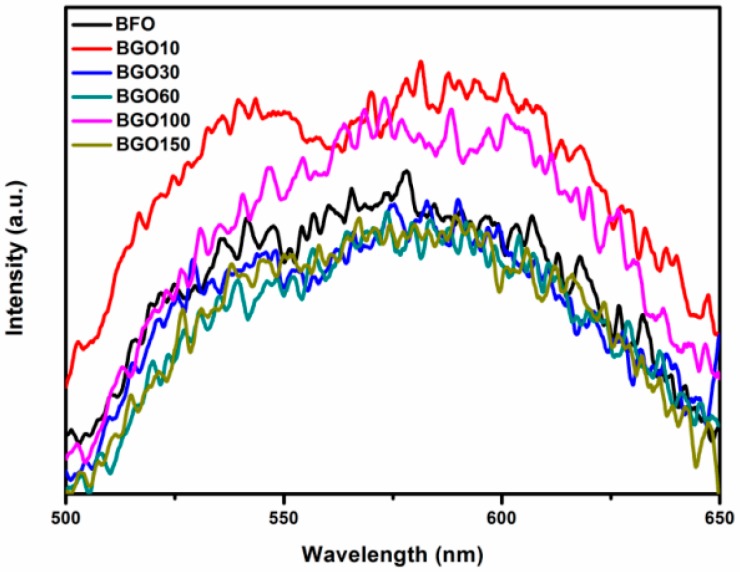
Photoluminescence spectroscopy of BFO and BGO samples.

Photoluminescence (PL) spectroscopy can be used as an effective probe to analyze the recombination rate of the electron hole pairs in the semiconductors. As shown in Figure 8, the emission intensity of the PL spectra for the BGO60 composites is lower than that of pure BFO, which reveals the decrease of the recombination rate of photogenerated electron hole in BFO/RGO composite photocatalysts. The close contact between BFO and RGO enables photogenerated carriers to migrate at the interface, thereby reducing the recombination rate of electron-hole pairs, which will effectively enhance the photocatalytic performance of photocatalysts.

### 3.3. Photocatalytic Activity

**Figure 9:**
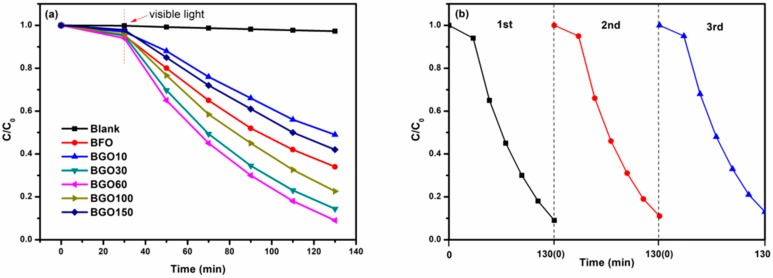
(**a**) Photocatalytic degradation efficiencies of methylene blue (MB) as a function of irradiation time under visible light for BFO and BGO samples; (**b**) Stability test of photocatalytic degradation of MB by BGO60 under visible light irradiation.

The photocatalytic performance of BFO and BGO samples are investigated by the degradation of methylene blue (MB) aqueous solution under the simulated visible light irradiation. The photo-degradation efficiency of organic molecules could be determined by the equation: Degration (%) = (C − C_0)_/C × 100. Figure 9a shows the photocatalytic degradation efficiency characterized by BFO and BGO composites. Under the irradiation of visible light, MB solution is almost completely degraded by BGO60 within 100 min, and the degradation rate is 92%. Compared with the pure BFO (34%), the photocatalytic degradation efficiency is enhanced obviously. BGO30, BGO100 and BGO150 also exhibited excellent photocatalytic properties, and the degradation rates were 86%, 78%, and 66%, respectively. In the blank sample measurements, the MB solution degraded 2% within 130 min, which should be due to the slight thermal catalytic effect. It was obvious that the photocatalytic performances of BGO composites were greatly affected by the content of GO. With the increase of GO, the photodegradation efficiency of BGO composites first increased then decreased. Among them, BGO60 showed the best photocatalytic activity, which was about 2.7 times than that of BFO. With the increase of GO content, the size of BFO particles decreased, resulting in an increase in contact area with RGO. At the same time, the ability of light absorption and effective photo-generated charge separation and transfer were also promoted, and the photocatalytic activity was enhanced accordingly. However, as GO continues to increase, the excess amount of RGO will act as a recombination of the photo-generated electron-hole, thereby resulting in a decline in the photocatalytic activity. Based on the experimental results, BGO60 was considered to have an optimal value of GO content. Remarkably, due to weak light absorption and larger particle size, the number of photo-generated carriers moving to the surface are less and easily recombine, resulting in poor photocatalytic performance of BGO10, this was also associated with the results in the PL spectrum. Additionally, the stability of the BGO60 photocatalyst was investigated in three repeating cycles, as shown in Figure 9b. There was a slight decrease (3%) in photocatalytic efficiency after three recycle times, which may be due to the loss of catalyst in cycling experiments, and the good cycle stability of the photocatalyst was proved.

Compared with pure BFO, the photocatalytic performance of the synthesized BFO/RGO composites have been obviously improved. The proposed mechanism of enhanced photocatalytic activity can be attributed to the following three aspects: (1) Enhanced optical absorption. In our study, the BGO sample exhibited excellent optical absorption in both ultraviolet and visible region. At the same time, the reduced band gap makes more absorbed photons form electron-hole pairs, resulting in more reactive species on the catalyst surface and participating in the photocatalytic reaction; (2) Increased contact area with pollutants. Graphene has a large and smooth graphite layer, which produces π-π interaction. It is easy to adsorb organic pollutants with π electrons [44]. It has good adsorption effect on methylene blue through the π-π and hydrogen bonds. A large number of RGO nanosheets attach to the surface of the BFO particles, resulting in an increased contact area between photocatalyst and organic pollutants; (3) A decrease in the recombination of the electron-hole pairs. The close contact between BFO and RGO leads to the transport of photogenerated carriers at the interface, and a semiconductor heterostructure may be formed locally [45]. It is believed that the delocalized conjugated structure and superior electrical conductivity of RGO facilitate the photogenerated electrons transferring from the conduction band of BFO to RGO, consequently suppressing the photogenerated electron-hole recombination, and eventually leading to the enhanced photocatalytic performance; (4) The affinity of graphene to dye molecules. RGO can in fact act as an entity to increase the affinity to the molecules surrounding it and attract them near the grafted BFO photocatalyst [46].

## 4. Conclusions

In summary, BFO/RGO composite photocatalysts have successfully been synthesized by a facile hydrothermal treatment. The XRD, SEM, Raman, and XPS characterizations confirm that graphene oxide was reduced in hydrothermal process and compounded with BFO. The optical properties show that the introduction of RGO can effectively enhance the optical absorption properties and reduce the recombination of the electron hole pairs. Compared with pure BFO, the photocatalytic performance of BGO composites is enhanced for degradation of MB solution under visible light irradiation, and the result shows that the optimal amount of GO in the composites is 60 mg (BGO60). The enhanced photocatalytic activity of the BGO composite photocatalysts should put down to the enhanced optical absorption, increased contact area with pollutants, and the decreased recombination of photogenerated electron-hole pairs. More importantly, BGO composites have the potential to be a highly efficient visible light response photocatalyst for wastewater treatment and hydrogen production.
